# A quantitative RT-PCR platform for high-throughput expression profiling of 2500 rice transcription factors

**DOI:** 10.1186/1746-4811-3-7

**Published:** 2007-06-08

**Authors:** Camila Caldana, Wolf-Rüdiger Scheible, Bernd Mueller-Roeber, Slobodan Ruzicic

**Affiliations:** 1 Max-Planck Institute of Molecular Plant Physiology, Am Mühlenberg 1, 14476 Potsdam-Golm, Germany; 2 University of Potsdam, Institute of Biochemistry and Biology, Karl-Liebknecht-Straße 24-25, Haus 20, 14476 Potsdam-Golm, Germany

## Abstract

**Background:**

Quantitative reverse transcription – polymerase chain reaction (qRT-PCR) has been demonstrated to be particularly suitable for the analysis of weakly expressed genes, such as those encoding transcription factors. Rice (*Oryza sativa *L.) is an important crop and the most advanced model for monocotyledonous species; its nuclear genome has been sequenced and molecular tools are being developed for functional analyses. However, high-throughput methods for rice research are still limited and a large-scale qRT-PCR platform for gene expression analyses has not been reported.

**Results:**

We established a qRT-PCR platform enabling the multi-parallel determination of the expression levels of more than 2500 rice transcription factor genes. Additionally, using different rice cultivars, tissues and physiological conditions, we evaluated the expression stability of seven reference genes. We demonstrate this resource allows specific and reliable detection of the expression of transcription factor genes in rice.

**Conclusion:**

Multi-parallel qRT-PCR allows the versatile and sensitive transcriptome profiling of large numbers of rice transcription factor genes. The new platform complements existing microarray-based expression profiling techniques, by allowing the analysis of lowly expressed transcription factor genes to determine their involvement in developmental or physiological processes. We expect that this resource will be of broad utility to the scientific community in the further development of rice as an important model for plant science.

## Background

Various high throughput techniques that allow the accurate quantification of expression levels (transcript abundance) of hundreds or thousands of genes are currently available [1]. Commonly, cDNA- and oligonucleotide-based microarrays are used to measure transcripts at a genome-wide scale [1]. However the usefulness of these is often limited by their sensitivity and accuracy, particularly for low-abundance transcripts. In contrast, quantitative reverse transcription – polymerase chain reaction (qRT-PCR or real-time RT-PCR) allows even weakly expressed genes to be accurately quantified [2]. Thus, whilst array-based hybridisation typically allows the detection of one transcript per cell [3,4], qRT-PCR can detect one transcript per 1000 cells [5]. Recent improvements in qRT-PCR methodology have eliminated many of the initial problems that were associated with quantitative gene expression studies, such as those arising from alternative splicing events [6]. Despite such developments qRT-PCR is mostly used to detect relatively small numbers of genes.

Transcription factors (TFs) are proteins (*trans*-acting factors) that enhance or repress gene expression through their binding to specific DNA sequences (*cis*-acting elements) in the promoters of their target genes. The functional characterization of TFs is crucial for the reconstruction of transcriptional regulatory networks controlling developmental and physiological processes such as growth, organ formation and the response to hormonal or environmental stimuli [7,8]. Transcription factor genes represent a sizable fraction of the genomes of all eukaryotic organisms, including higher plants [7]. Analysis of the rice genome [9,10] indicated that approximately 2.6% of the identified genes encode TFs [10]. Currently, the functional analysis of TFs in monocotyledonous species lags considerably behind that of the model dicotyledonous species *Arabidopsis thaliana*.

Microarray expression profiling in rice has not been widely reported with relatively few publicly available data. Studies using qRT-PCR have also not been widely reported, have focussed on small groups of genes, and in many cases were only used to confirm expression changes from microarray experiments. Therefore, the utility of qRT-PCR as a high-throughput method in rice has not been investigated.

To facilitate the analysis of rice TFs we have recently established a database [11,12]. The coding sequences of more than 2500 identified rice TFs were used to design primers for a large-scale qRT-PCR platform. The comparative analysis of several rice varieties and tissues described here has confirmed the broad applicability of the platform.

## Results and Discussion

### Primer design

Analysis of the rice genome indicated that 21% of all genes give rise to alternatively spliced transcripts [13]. In the case of TFs, splice variants can affect the architecture of the DNA-binding domain and often show tissue-specific expression patterns [14]. To distinguish between such variants, splice variant-specific primer pairs were designed for the 5.7 % of all TF loci (131 TF loci) where this was possible. In total, primer pairs for 2508 gene models derived from 2306 loci were designed (Additional file 1). The design of primers followed a set of stringent criteria, as generally suggested in qRT-PCR protocols (e.g. Primer Express Software v2.0 Application Manual, Applied Biosystems). To minimize the risk of amplifying contaminating genomic DNA, primers spanning at least one exon-exon junction, or annealing to different exons, were designed where possible (56% of predicted gene models). However, 35% of genes contained no introns. The specificity of each primer was confirmed by comparing its sequence with all predicted rice coding sequences (CDS) using the BLASTN tool at TIGR [15] to ensure that at least one primer of each pair targets a unique site within the set of predicted rice CDS.

### RNA sampling and control for genomic DNA contamination

RNA was initially extracted using a phenol-based method from two different tissues (root and shoot) of four rice cultivars, three of which were *indica *cultivars (Cham, DR2 and Lua man) and the fourth was a *japonica *cultivar (Nipponbare). This protocol (e.g. as described by Czechowski *et al. *and Jain *et al. *[5,16]) gave satisfactory total RNA yield, but the RNA quality was too low for the synthesis of high-quality cDNA [data not shown] [17]. We therefore used the guanidinium thiocyanate-based RNeasy Plant Mini Kit (Qiagen, Hilden, Germany) [18]. Total RNA extraction was straightforward and provided RNA with high yield and quality from both root and shoot tissue (45–75 μg total RNA/100 mg fresh weight).

RNA preparations are usually contaminated with low amounts of genomic DNA, which can result in non-specific amplification [19]. The manufacturer's recommended on-column DNAse treatment was not sufficient to remove interfering genomic DNA. Therefore, we performed a second DNAse incubation on the isolated RNA to eliminate detectable genomic DNA contamination. Genomic DNA was detected by qRT-PCR using 0.125 μg of isolated RNA as template, and three different primer pairs annealing to intergenic regions of chromosomes 1 (AP003727, positions 7366–7426) and 7 (AP006456, positions 27334624–27334684), and an intron of the gene Os01g01840. It is important to select more than a single genomic region to assess genomic DNA contamination because chromosomal sites can be differentially accessible to DNAse I. Omitting the second DNAse digestion always resulted in the amplification of some, but not all, of these genomic regions (data not shown). In most cases the C_T _values obtained were >30. Synthesis of cDNA from the isolated RNA was only performed when all three genomic control amplifications scored negative.

**Figure 1:**
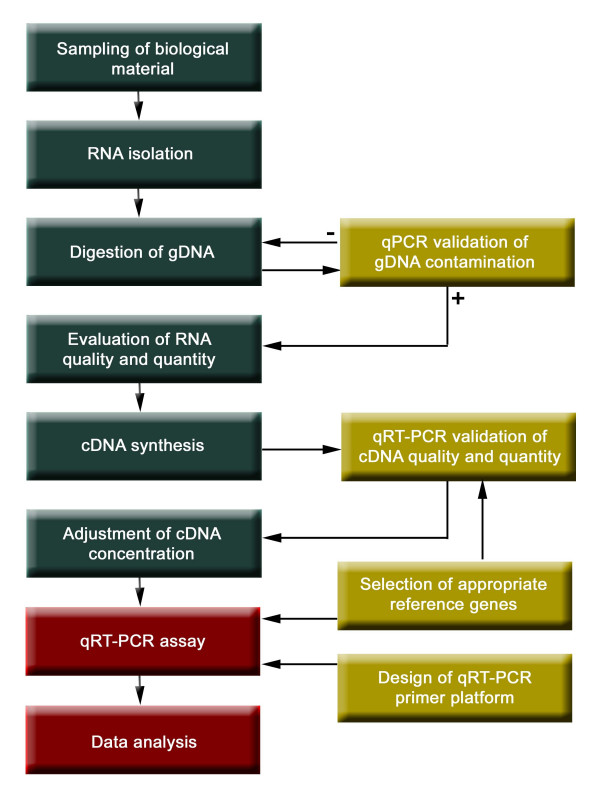
**Flow-chart of an optimised protocol for qRT-PCR in large-scale expression profiling experiments**. The protocol implements multiple optimised and control steps including RNA isolation, digestion of genomic DNA (gDNA), evaluation of cDNA quality, primer design and data analysis. The absence of gDNA was confirmed by quantitative RT-PCR (qRT-PCR) with primer pairs targeting various non-coding regions. The quality of the cDNA was tested using different reference genes, as outlined in the text.

We chose a two-step qRT-PCR protocol where reverse transcription and PCR-mediated cDNA amplification are carried out in subsequent steps in separate tubes. The two-step protocol is preferred when SYBR Green is used as a detection dye because it diminishes unwanted primer dimer formation [20]. The reverse transcriptase reaction was primed with oligo-(dT) instead of random-sequence primers because the latter preferentially selects for more abundant mRNA species and not for transcripts of weakly expressed genes, such as many TF genes. A flowchart of the protocol is shown in Figure 1.

### Reaction specificity

All 2508 primer pairs were checked by qRT-PCR using cDNAs synthesised from the roots of the rice cultivars, Cham and DR2 kept under control and salt stress (100 mM NaCl) conditions. Additionally, shoot cDNA from DR2 was used to test a smaller set of primer pairs targeting 192 TF genes. Melting curve analysis was performed for all PCR products to confirm the occurrence of specific amplification peaks and the absence of primer-dimer formation. Finally, all 2508 PCR products from the control DR2 root sample were run on 4% agarose gels and photographically documented (available upon request).

Approximately 3% of all TF genes analysed (i.e. 73 out of 2508 genes) did not yield detectable PCR amplicons, indicating no or weak expression under the employed conditions. In most cases (57 TF genes) at least one primer spanned an exon-exon junction thus precluding tests for priming efficiency on genomic DNA template. Only 2.5% of all reactions (61 TF genes) yielded unspecific PCR products as indicated by multiple or incorrectly sized amplicons.

### Primer efficiency

Although the fluorescence during qRT-PCR is primarily determined by the starting abundance of a given cDNA template, its increase during the run is strongly affected by the amplification efficiency. When this efficiency is 100%, the amount of a cDNA targeted by a given primer pair is doubled in every PCR cycle of the exponential phase. However, the priming site, the sequence specificity of the primer, and the unwanted formation of primer-dimers are factors that can significantly affect the efficiency of individual PCR reactions. We used the LinRegPCR software [21] to determine the PCR efficiency of each primer pair of the TF qRT-PCR platform, taking into account all amplification profiles obtained (27588 in total). Firstly, the correlation coefficients (R) assigned to efficiency values were used to evaluate the amplification curves and all reactions with an R < 0.990, reflecting low-quality amplifications, were excluded from further analyses. Subsequently, average PCR efficiencies were computed for each individual primer pair across all analyzed samples. This showed that 8% of the 2508 primer pairs (200 TF genes) displayed PCR efficiencies greater than 1.90, and 87% of the primer pairs (2182 TF genes) had efficiencies of 1.51–1.90. Only 5% of the primer pairs (125 TF genes) had PCR efficiencies with mean values below 1.4, and these mostly represented reactions that lacked detectable fragment amplification (C_T _> 40) or that generated unspecific PCR products. The efficiencies of reference genes tested in this work ranged from 1.73 to 1.96 (see below; Table 1).

**Table 1:** Selected reference genes for rice and corresponding primer pair information.

**Locus identifier**	**Gene name**	**Primer sequence F/R [5'-3']**	**Amplicon length [bp]**	**Amplicon T_m _[°C]**	**PCR efficiency**
Os03g50890	Actin	CTCCCCCATGCTATCCTTCG TGAATGAGTAACCACGCTCCG^[S]^	91	81	1.9
Os05g36290*	Actin1	ATCCTTGTATGCTAGCGGTCGA ATCCAACCGGAGGATAGCATG^[D]^	118	80	1.9
Os01g59150*	β-Tubulin	GGAGTCACATGCTGCCTAAGGTT TCACTGCCAGCTTACGGAGG^[S]^	64	80	1.96
Os06g11070	Expressed protein	AGGCTGGTCGAGGAGTCCAT TTCTCCTCCCTAGCGAACACCT^[D]^	101	85	1.83
Os03g55270	TIP41-like	GTTTGGATGAACCCCGCAA GGCAACAAGGTCAATCCGATC^[S]^	62	77.5	1.81
Os08g19610	Cyclophilin	CCACCATCACAGATCGGATCTT GCGGTCAGAGCGAAAGTAGCTA^[S]^	65	84.2	1.73
Os03g08020*	Elongation factor 1α	GTCATTGGCCACGTCGACTC TGTTCATCTCAGCGGCTTCC^[S]^	118	83.5	1.85

* Primer pair recognizes all splicing variants. Location of forward and reverse primer in the same exon [S] or in different exons [D].

**Figure 2:**
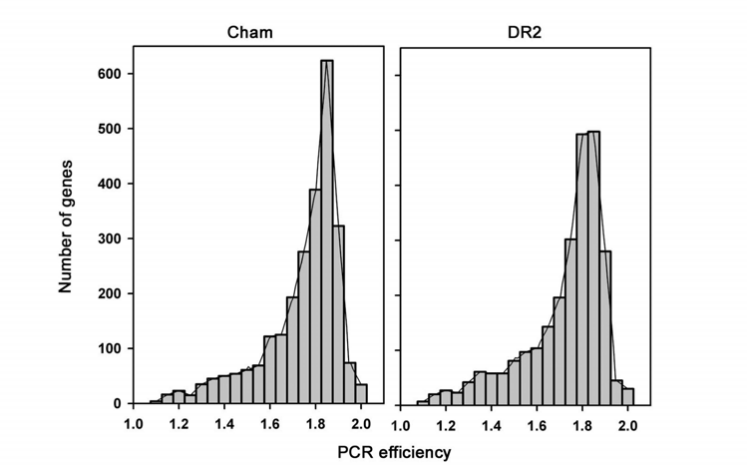
**Distribution of calculated PCR efficiencies as determined for two *indica *rice cultivars**. The two distributions were compared using a Kolmogorov-Smirnov test and were found to be significantly different at p = 0.001. Non-parametric comparison of mean values (Mann-Whitney U test) confirmed the presence of statistically significant differences at p = 0.000001. Transformation to expression values revealed that the slightly different PCR efficiencies could lead to a mean difference of maximal 0.3, when the fold change was expressed as log_2_. Individual primer pairs can thus exhibit slight differences for their target genes in different cultivars. However, this does not significantly affect the overall applicability of the primer platform for expression profiling experiments (Caldana *et al*., manuscript in preparation).

The TF qRT-PCR platform was developed primarily using the nuclear genome sequence of the *japonica *cultivar Nipponbare (TIGR annotation) [15], which might affect its general applicability for experiments involving *indica *cultivars. SNP (single nucleotide polymorphism) variation among the two rice subspecies was found to be low (<0.4%) [22], and these mostly occur in intergenic regions which contain approximately four times more SNPs than occur within genes [23]. SNPs may affect the comparative analysis of gene expression levels in qRT-PCR experiments. Therefore, to investigate variation of primer specificity between the *indica *varieties Cham and DR2, PCR efficiencies were separately computed in a cultivar-specific manner for all gene models. Although individual primer pairs of the TF primer platform can exhibit slight differences for their target genes in different varieties/cultivars (Figure 2), this did not significantly affect the overall applicability of the platform for expression profiling experiments, a finding we have also validated in independent experiments (Caldana *et al.*, manuscript in preparation).

### Accuracy and precision of real-time PCR

**Figure 3:**
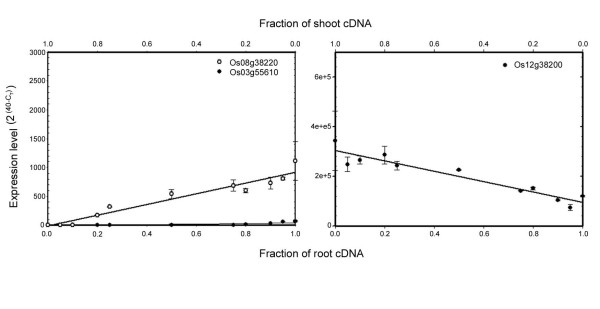
**Linearity and sensitivity of qRT-PCR**. cDNA derived from root or shoot RNA was mixed in different ratios (as indicated; total amount of cDNA was 1 ng) and used as template to test transcript abundance of three selected genes (Os03g55610, Os08g38220, and Os12g38200) via qRT-PCR. A linear relationship between root (or shoot) cDNA and expression level of the various genes was observed. Symbols in both panels represent the mean ± SD (*n *= 3).

To determine the sensitivity and accuracy of the rice qRT-PCR platform, as reported for other platforms [5], we performed additional experiments. Firstly, we assessed the linearity, sensitivity and accuracy using three weakly expressed genes with preferential expression in either shoots (Os12g38200) or roots (Os03g55610 and Os08g38220). Shoot- and root-derived cDNAs were mixed in different ratios (see Figure 3) and transcript abundance of the three genes was determined by qRT-PCR. Linearised values were calculated as 2(40−CT)
 MathType@MTEF@5@5@+=feaafiart1ev1aaatCvAUfKttLearuWrP9MDH5MBPbIqV92AaeXatLxBI9gBaebbnrfifHhDYfgasaacH8akY=wiFfYdH8Gipec8Eeeu0xXdbba9frFj0=OqFfea0dXdd9vqai=hGuQ8kuc9pgc9s8qqaq=dirpe0xb9q8qiLsFr0=vr0=vr0dc8meaabaqaciaacaGaaeqabaqabeGadaaakeaacqaIYaGmdaahaaWcbeqaaiabcIcaOiabisda0iabicdaWiabgkHiTiabboeadnaaBaaameaacqqGubavaeqaaSGaeiykaKcaaaaa@34C2@, where C_T _represents the threshold cycle and directly reflects template abundance. A linear relationship was observed in all three cases, suggesting that, as previously reported for *Arabidopsis *[5], qRT-PCR was sufficiently accurate in rice even for low-abundant transcripts. These relationships were highly significant with *R*^2 ^values (*coefficient of determination*) of 0.61, 0.94, and 0.90 for Os03g55610, Os08g38220, and Os12g38200, respectively (Figure 3).

We also tested the experimental reproducibility of the rice qRT-PCR platform. Czechowski *et al*. [5] demonstrated qRT-PCR precision by analysing intra-assay variation using the same pool of cDNAs and inter-assay variation using two different pools of cDNAs synthesised from the same batch of RNA, obtaining *R*^2 ^values of 0.99 and 0.95, respectively. Here, we wanted to determine the variation between different biological replicates which encompasses both technical and biological variation. Using cDNA synthesised from DR2 harvested in three independent experiments we measured the expression of 201 TF genes. The ΔC_T _(C_T_gene of interest _- C_T_reference gene_) was calculated and the precision of the assay was assessed using the *coefficient of variation *(CV). Despite the fact that the majority of gene models tested (111 genes) displayed an extremely low expression level (C_T _> 35), the obtained mean CV was 14%. This is in good agreement with published expression data for human keratinocyte subclones, in which a CV of 18% was found for genes with a C_T _> 30 [24]. In contrast, CV values are generally higher in microarray-based analyses of genes with such low expression, indicating a lower reproducibility [25]. These findings underscore the advantage of qRT-PCR as an alternative and often superior tool for expression profiling studies, especially for the investigation of genes with low expression level.

### Selection of reference genes for qRT-PCR in rice

Generally in qRT-PCR, transcripts of stably expressed genes, also called reference genes, are employed for data normalisation. In rice, previous publications have suggested *18S-rRNA*, *GADPH*, *UBI5 *and *EF-1α *as good reference genes [26,16]. To identify the most suitable reference genes in rice we initially selected nine candidates: *18S-rRNA*, *ubiquitin *(*UBQ*), *actin *(*ACT*), *actin1 *(*ACT1*), *β-tubulin *(*TUB*), *cyclophilin *(*CYC*), *elongation factor 1α *(*EF-1α*), which are commonly used house-keeping genes in plants, and *expressed protein *(*EP*) and *TIP41-like protein *(*TIP41*) found to be good reference genes in *Arabidopsis *[27]. *UBQ *(Os01g45420), considered as a stable house-keeping gene in various plant species [27,16], had unstable expression across three different rice cultivars (data not shown), and was excluded from further studies. Although the abundance of 18S-rRNA remained constant in different rice cultivars and physiological conditions (data not shown), we did not consider it further as a suitable reference for our analyses, primarily because it requires the use of random hexamers instead of oligo(dT) as primers for the reverse transcriptase.

An overview of the remaining seven reference genes is given in Table 1. *CYC*, *EP *and *TIP41 *are expressed at low, *TUB*, *ACT *and *ACT1 *at intermediate, and *EF-1α *at high levels, respectively. Their expression stability was measured by qRT-PCR in a set of 11 different cDNA samples (Additional file 2), and calculated using the *gene expression stability measure *(M) implemented in the geNORM software [28]. This determines the stability of a reference gene, taking into account the *average pair-wise variation *(V) of that gene in comparison to all other reference genes tested. Assuming that the difference of gene expression level of two ideal control genes is the same in all experimental conditions (tissues, treatments) compared, the lower a genes M value is, the more stably it is expressed (suggested limit of M<1.5).

**Figure 4:**
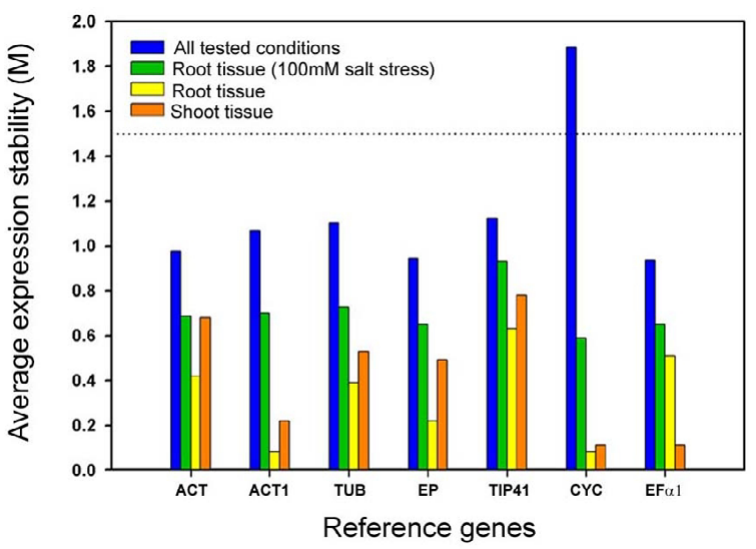
**Expression stability of selected reference genes as calculated by geNORM**. The data sets used for the calculation of the *average expression stability *(M) values are provided in Additional file 2. 'All tested conditions' includes all probes tested (different cultivars, tissues and physiological conditions); 'Root tissue (100 mM salt stress)' includes all root samples of salt stress-treated plants; 'Root tissue' and 'Shoot tissue' include samples of non-stressed tissues of different cultivars. A lower M value indicates more stable expression. According to the geNORM manual, genes with M values >1.5 are not suitable reference genes for the selected conditions. Gene codes and primers used for the qRT-PCR experiments are given in Table 1.

To follow the relationship between expression stability and experimental condition, geNORM was run with different input data (Figure 4). The first experimental series used the 11 cDNA samples. Except for *CYC*, which exhibited an M value of 1.88, all genes displayed values below the threshold of 1.5, proving their stability. *EF-1α *and *EP *had the most stable expression and are consequently the best overall reference genes. We also compared expression stability using shoot or root tissue of the different rice cultivars (Figure 4). Although all genes had M values below 1.5, *TIP41 *was the least stably expressed gene in both tissues. This is in contrast to the stable expression of the orthologous gene from *Arabidopsis *(At4g34270) across many different tissues [27]. Together with our data from *UBQ*, this highlights the need to test the suitability of a given gene even if its orthologue in another species is an adequate reference gene. We also tested the affect of salt stress on the expression stability of the selected genes. As in the tissue experiment, all genes displayed satisfactory M values and the best ranked genes were *ACT1 *and *CYC*, followed by *EF-1α*. However, the weakly expressed gene *CYC *was the least stable when more diverse conditions were used. Therefore, considering all the experimental categories, *ACT1 *and *EF-1α *were the most stable reference genes. Additionally, *EP*, similar to its *Arabidopsis *orthologue (At4g33380) [27], showed good stability in all experiments. Generally, we recommend including more than a single reference gene in each qRT-PCR experiment. Conveniently, the geNORM software also calculates the pair-wise variation (V), which indicates the optimal number of reference genes to be analysed in a given experiment.

## Conclusion

A qRT-PCR platform for multi-parallel expression profiling of more than 2500 rice TF genes has been established. It complements existing microarray-based profiling technologies, which are generally not well suited for reliable analysis of weakly expressed genes including TFs. This resource is available for the scientific community to use for their own experiments.

## Methods

### Plant material

The three rice (*Oryza sativa *L. ssp. *indica*) cultivars (Cham, DR2 and Lua man) were obtained from the Institute of Biotechnology (Hanoi, Vietnam). Additionally, a Nipponbare (*Oryza sativa *L. spp. *japonica*) cultivar provided by the International Rice Research Institute (Manila, Philippines) was used. The plants were grown in hydroponic culture [29] under a day-length of 12 h at 26/22°C (day/night), 70% humidity and 700 μmol m^-2 ^s^-1 ^light intensity. Roots and shoots were harvested three weeks after germination. Salt-stressed plants (Cham, DR2, and Lua man) were harvested 30 min or 3 h after the application of 100 mM NaCl, with control samples harvested in parallel.

### RNA extraction, DNAse I digestion and cDNA synthesis

The roots and shoots of three biological replicates with five plants each were pooled to isolate total RNA using the RNeasy Plant Mini Kit (Qiagen, Hilden, Germany) according to the manufacturer's protocol. During the extraction, the first on-column DNAse I (Qiagen) digestion was carried out. A second DNAse I (Roche, Mannheim, Germany) digestion was performed on a total of 60 μg of total RNA according to the manufacturer's instructions. Absence of genomic DNA contamination was subsequently confirmed by qRT-PCR using three different primer pairs. The primers were designed to amplify two intergenic regions (AP003727, positions 7366 – 7426: forward 5'-AGAGAAGACCGCCATGTTGG-3' and reverse 5'-CTGGCACCACAAAAACAATGAC-3'; and AP006456, positions 27334624 – 27334684: forward 5'-TATCCACTCGACAGGACGTGC-3' and reverse 5'-CCGGCAGGCAAGCTACTAGAC-3'), and an intron sequence of the gene Os01g01840 (forward 5'-TAGAGAGTTCGATCTTGCGCG-3' and reverse 5'-CGGCCCATTCAATGAAGTCTT-3'). RNA integrity was checked on 1% (w/v) agarose gels and the concentration measured before and after DNAse I digestion. cDNA was synthesized from 5 μg of total RNA using Superscript™ III reverse transcriptase (Invitrogen, Karlsruhe, Germany), according to the manufacturer's instructions. The efficiency of cDNA synthesis was estimated by qRT-PCR using *β-tubulin *(Os01g59150, forward primer 5'-GGAGTCACATGCTGCCTAAGGTT-3' and reverse primer 5'-TCACTGCCAGCTTACGGAGG-3').

### Design and validation of qRT-PCR primers

Transcription factor sequences were extracted from version 1 of the Rice Transcription Factor Database [11,12] and used to establish the rice TF qRT-PCR platform. The set of 2508 gene models corresponding to 2306 loci of confirmed and putative transcription factors was subsequently used to design primers for ca. 500 genes. The remaining 2000 primer pairs were designed by MWG Biotech AG (Ebersberg, Germany). A standard set of reaction conditions and a set of stringent criteria were used as follows: T_m _of 60°C ± 2°C, PCR amplicon length of 60 to 150 bp, primer length of 20 ± 5 bp, and a guanine-cytosine content of 45 to 55%. If gene structure allowed, at least one primer was designed to cover an exon-exon junction. The specificity of the primer pair sequence was checked against Version 2 of the rice transcripts (CDS) from the TIGR Rice Database [15] using the BLAST programme. The EXPECT threshold (statistical significance) was set to 1000, as suggested when searching for short, nearly exact matches [30]. The specificity of the amplicons was checked by qRT-PCR dissociation curve analysis and electrophoresis of PCR products on 4% agarose gels. The efficiencies (E) of the polymerase chain reactions were estimated using the LinRegPCR software [21] (Additional file 1).

### Selection of reference genes

Potential reference genes were chosen based on published data for rice and other plant species and rice orthologues were identified using the TBLASTN programme [15]. The gene models used were: *actin *(Os03g50890), *actin1 *(Os05g36290), *β-tubulin *(Os01g59150), *expressed protein *(Os06g11070), *TIP41-like protein *(Os03g55270), *cyclophilin *(Os08g19610) and *elongation factor 1α *(Os03g08020). Primer design followed the same criteria as described and details are given in Table 1.

### Quantitative RT-PCR conditions and analysis

PCR reactions were conducted in an ABI PRISM 7900 HT sequence detection system (Applied Biosystems). A 5 μl reaction containing 0.5 μl of cDNA (1.25 ng/μl), 200 nM of each gene-specific primer and 2.5 μl of SYBR Green master mix (Applied Biosystems Applera, Darmstadt, Germany), was used to monitor double-strand DNA synthesis. The qRT-PCR reactions were carried out following the recommended thermal profile: 50°C for 2 min, 95°C for 10 min, followed by 40 cycles of 95°C for 15 s and 60°C for 1 min. After 40 cycles, the specificity of the amplifications was tested by heating from 60°C to 95°C with a ramp speed of 1.9°C min^-1^, resulting in melting curves. Data analysis was performed using SDS 2.2.1 software (Applied Biosystems). All amplification curves were analysed with a normalized reporter (R_n_: the ratio of the fluorescence emission intensity of SYBR Green to the fluorescence signal of the passive reference dye) threshold of 0.2 to obtain the C_T _values (threshold cycle). The reference control genes were measured with four replicates in each PCR run, and their average C_T _was used for relative expression analyses. TF expression data were normalized by subtracting the mean reference gene C_T _value from their C_T _value (ΔC_T_). The Fold Change value was calculated using the expression 2−ΔΔCT
 MathType@MTEF@5@5@+=feaafiart1ev1aaatCvAUfKttLearuWrP9MDH5MBPbIqV92AaeXatLxBI9gBaebbnrfifHhDYfgasaacH8akY=wiFfYdH8Gipec8Eeeu0xXdbba9frFj0=OqFfea0dXdd9vqai=hGuQ8kuc9pgc9s8qqaq=dirpe0xb9q8qiLsFr0=vr0=vr0dc8meaabaqaciaacaGaaeqabaqabeGadaaakeaacqaIYaGmdaahaaWcbeqaaiabgkHiTiabfs5aejabfs5aejabboeadnaaBaaameaacqqGubavaeqaaaaaaaa@33ED@, where ΔΔC_T _represents ΔC_Tcondition of interest _- ΔC_T control_. The obtained results were transformed to log_2 _scale.

### Dilution experiments

Mixtures of root and shoot cDNA were prepared as described by Czechowski *et al. *[5] to give the ratios indicated in Figure 3. Primer pairs for two root-specific genes (Os03g55610, 5'-TACCCCACCCAACACCATCATCTG and 5'-TGAGAAGAAGGCAAAGGCAGTGAG; Os08g38220; 5'-GGGGTTTCCAACTACACCCGATGA and 5'-TCACCATATACCCCACGCGCAA) and one shoot-specific gene (Os12g38200, 5'-TGGTTTTCTCCTCGCTTCCAATCT and 5'-TGCTGCTGCATCTGAGTCCAATT) were used in qRT-PCR experiments.

### Determination of reference gene expression stability

To analyse the expression stability of the selected reference genes, the geNORM v.3.4 software was used as described by Vandesompele *et al. *[28]. The measured C_T _values were transformed so the highest expression of each reference gene is equal to 1 and its expression in all other conditions is relative to this value (as suggested by the geNORM manual). The gene expression stability (M) was calculated and the most stable control genes were determined.

## Competing interests

The author(s) declare that they have no competing interests.

## Authors' contributions

CC performed the experimental work and drafted the manuscript. WRS helped to outline the project. BMR and SR designed and coordinated the project and wrote the manuscript. Overall supervision was provided by BMR.

## Supplementary Material

Additional file 1 Complete list of TF genes, primer data and corresponding PCR efficiencies. The TF primer platform was established based on the data for the gene models according to the Version 2.0 of TIGR Rice genome annotation. The corresponding new identifiers (Version 5.0) are also presented. The ranking of primer pairs taking into account experimental data (Cat column) was done as follows: 1, specific amplification; 2, no amplification; 3, non-specific amplification. 'Primer Location' indicates the sites within genes selected for primer design: J, at least one of the primers extends over an exon-exon junction; D, the primers are located in different exons; S, both primers are located within same exon. Click here for file

Additional file 2 cDNA samples used for the validation of the reference genes. Click here for file
