# Supplementary material for: A quantitative RT-PCR platform for high-throughput expression profiling of 2500 rice transcription factors
Source: Plant Methods. 2007 Jun 8;3:7. doi: 10.1186/1746-4811-3-7 (PMC1914063; doi:10.1186/1746-4811-3-7)
Supplement: Additional file 2 — cDNA samples used for the validation of the reference genes. [file 1746-4811-3-7-S2.doc]

### Additional file 2. cDNA samples used for the validation of the reference genes.

| **cDNA pool** | **Plant material** | **Rice cultivar** |
| --- | --- | --- |
| 1 | 3 weeks old, root | *ssp. indica* cv Cham |
| 2 | 3 weeks old, root, 30 minutes 100mM NaCl | *ssp. indica* Cham |
| 3 | 3 weeks old, root | *ssp. indica* DR2 |
| 4 | 3 weeks old, root, 3 hours 100mM NaCl | *ssp. indica* DR2 |
| 5 | 3 weeks old, root | *ssp. indica* Lua man |
| 6 | 3 weeks old, root, 3 hours 100mM NaCl | *ssp. indica* Lua man |
| 7 | 3 weeks old, shoot | *ssp. indica* DR2 |
| 8 | 3 weeks old, shoot | *ssp. indica* Cham |
| 9 | 3 weeks old, shoot | *ssp. indica* Lua man |
| 10 | 3 weeks old, root | *ssp. japonica* Nipponbare |
| 11 | 3 weeks old, shoot | *ssp. japonica* Nipponbare |
